# Supplementary material for: Inducing Magnetic Properties with Ferrite Nanoparticles in Resins for Additive Manufacturing
Source: Int J Mol Sci. 2023 Jul 24;24(14):11838. doi: 10.3390/ijms241411838 (PMC10380220; doi:10.3390/ijms241411838)
Supplement: Supplementary file 1 [file ijms-24-11838-s001.zip › ijms-2458056-supplementary.pdf]

Inducing Magnetic Properties with Ferrite Nanoparticles in Resins for Additive Manufacturing

Rocío Redón [1,*], Miriam D. Aviles-Avila [1], Leopoldo Ruiz-Huerta [1,2], Herlinda Montiel [1], Alex Elías-Zúñiga [2,3], Lucy-Caterine Daza-Gómez[1] and Oscar Martínez-Romero [2,3]

[1] Instituto de Ciencias Aplicadas y Tecnología, Universidad Nacional Autónoma de México, Cd. Universitaria, Coyoacán, Mexico City 04510, Mexico; leoruiz@unam.mx (L.R.-H.);

[2] National Laboratory for Additive and Digital Manufacturing (MADiT), Universidad Nacional Autónoma de México, Mexico City 04510, Mexico; oscar.martinez@tec.mx (O.M.-R.)

[3] Department of Mechanical Engineering and Advanced Materials, Institute of Advanced Materials for Sustainable Manufacturing, Tecnológico de Monterrey, Av. Eugenio Garza Sada Sur 2501, Monterrey 64849, Mexico

* Correspondence: rredon@unam.mx; Tel.: +52-5556228602 (ext. 1207)

Table S1. w/w % of the mechanochemical reactions between nanoparticles of barium ferrite ($BaFe_{12}O_{19}$) - Resin IRIX White and, between nanoparticles of cobalt ferrite ($CoFe_2O_4$) - Resin IRIX White.

Sample name	Sample content	Nanoparticles percentage [w/w %]
**A_0**	Resin IRIX White	0
**A_1**	$BaFe_{12}O_{19}$ and IRIX White resin	0.1
**A_3**	$BaFe_{12}O_{19}$ and IRIX White resin	0.3
**A_5**	$BaFe_{12}O_{19}$ and IRIX White resin	0.5
**A_7**	$BaFe_{12}O_{19}$ and IRIX White resin	0.7
**A_{10}**	$BaFe_{12}O_{19}$ and IRIX White resin	1.0
**B_1**	$CoFe_2O_4$ and IRIX White resin	0.1
**B_3**	$CoFe_2O_4$ and IRIX White resin	0.3
**B_5**	$CoFe_2O_4$ and IRIX White resin	0.5
**B_7**	$CoFe_2O_4$ and IRIX White resin	0.7
**B_{10}**	$CoFe_2O_4$ and IRIX White resin	1.0

Table S2. Percentages w%/w% of the mechanochemical reactions between nanoparticles of barium ferrite ($BaFe_{12}O_{19}$) - Resin Anycubic Green and, between nanoparticles of cobalt ferrite ($CoFe_2O_4$) - Resin Anycubic Green.

Sample name	Sample content	Nanoparticles percentage [w%/w%]
**C_0**	Resin Anycubic Green	0
**C_1**	$BaFe_{12}O_{19}$ and Anycubic Green resin	0.1
**C_3**	$BaFe_{12}O_{19}$ and Anycubic Green resin	0.3
**C_5**	$BaFe_{12}O_{19}$ and Anycubic Green resin	0.5
**C_7**	$BaFe_{12}O_{19}$ and Anycubic Green resin	0.7
**C_{10}**	$BaFe_{12}O_{19}$ and Anycubic Green resin	1.0
**C_{20}**	$BaFe_{12}O_{19}$ and Anycubic Green resin	2.0
**C_{30}**	$BaFe_{12}O_{19}$ and Anycubic Green resin	3.0
**C_{40}**	$BaFe_{12}O_{19}$ and Anycubic Green resin	4.0
**C_{50}**	$BaFe_{12}O_{19}$ and Anycubic Green resin	5.0
**C_{60}**	$BaFe_{12}O_{19}$ and Anycubic Green resin	6.0
**D_1**	$CoFe_2O_4$ and Anycubic Green resin	0.1
**D_3**	$CoFe_2O_4$ and Anycubic Green resin	0.3
**D_5**	$CoFe_2O_4$ and Anycubic Green resin	0.5
**D_7**	$CoFe_2O_4$ and Anycubic Green resin	0.7
**D_{10}**	$CoFe_2O_4$ and Anycubic Green resin	1.0
**D_{20}**	$CoFe_2O_4$ and Anycubic Green resin	2.0
**D_{30}**	$CoFe_2O_4$ and Anycubic Green resin	3.0
**D_{40}**	$CoFe_2O_4$ and Anycubic Green resin	4.0
